# Probiotic *Bacillus safensis* NPUST1 Administration Improves Growth Performance, Gut Microbiota, and Innate Immunity against *Streptococcus iniae* in Nile tilapia (*Oreochromis niloticus*)

**DOI:** 10.3390/microorganisms9122494

**Published:** 2021-12-01

**Authors:** Pei-Shan Wu, Chun-Hong Liu, Shao-Yang Hu

**Affiliations:** 1 Department of Biological Science and Technology, National Pingtung University of Science and Technology, Pingtung 912, Taiwan; wps606@gmail.com; 2 Department of Aquaculture, National Pingtung University of Science and Technology, Pingtung 912, Taiwan; chliu@mail.npust.edu.tw; 3 Research Center for Animal Biologics, National Pingtung University of Science and Technology, Pingtung 912, Taiwan

**Keywords:** *Bacillus* *safensis*, *Oreochromis niloticus*, probiotic, growth, innate immunity

## Abstract

Probiotics are considered ecofriendly alternatives to antibiotics as immunostimulants against pathogen infections in aquaculture. In the present study, protease-, amylase-, cellulase-, and xylanase-producing *Bacillus safensis* NPUST1 were isolated from the gut of Nile tilapia, and the beneficial effects of *B. safensis* NPUST1 on growth, innate immunity, disease resistance and gut microbiota in Nile tilapia were evaluated by feeding tilapia a basal diet or basal diet containing 10^5^ and 10^6^–10^7^ CFU/g for 8 weeks. The results showed that the weight gain, feed efficiency and specific growth rate were significantly increased in tilapia fed a diet containing 10^6^ CFU/g and 10^7^ CFU/g *B. safensis* NPUST1. Intestinal digestive enzymes, including protease, amylase and lipase, and hepatic mRNA expression of glucose metabolism and growth-related genes, such as *GK*, *G6Pase*, *GHR* and *IGF-1*, were also significantly increased in the 10^6^ CFU/g and 10^7^ CFU/g *B. safensis* NPUST1 treated groups. Immune parameters such as phagocytic activity, respiratory burst and superoxide dismutase activity in head kidney leukocytes, serum lysozyme, and the mRNA expression of *IL-1β*, *IL-8*, *TNF-α* and *lysozyme* genes were significantly induced in the head kidney and spleen of 10^6^ CFU/g and 10^7^ CFU/g *B. safensis* NPUST1 treated fish. The cumulative survival rate was significantly increased in fish fed a diet containing 10^6^ CFU/g and 10^7^ CFU/g *B. safensis* NPUST1 after challenge with *Streptococcus iniae*. Dietary supplementation with *B. safensis* NPUST1 improves the gut microbiota of Nile tilapia, which increases the abundance of potential probiotics and reduces the abundance of pathogenic pathogens. The present study is the first to report the use of *B. safensis* as a potential probiotic in aquaculture, and a diet containing 10^6^ CFU/g *B. safensis* NPUST1 is adequate for providing beneficial effects on growth performance and health status in tilapia.

## 1. Introduction

Global aquaculture has been considered the fastest growing method for producing animal protein to satisfy the increasing global food demand. According to Food and Agriculture Organization (FAO) 2020 statistics, aquaculture now accounts for more than 50% of the total fish produced for human consumption [1]. Nile tilapia (*Oreochromis niloticus*) is one of the most popular fish species worldwide due to its properties including fast growth, high tolerance to environmental stress and high marketability. Tilapia has been farmed in over 100 countries worldwide and is an important source of income in several developing countries. Intensive culture is commonly used in tilapia aquaculture to achieve high production yields in places with limited farmland. However, the high incidence of bacteria induced diseases due to deteriorated water quality resulting from intensive culture has become a major issue that impedes the sustainability of aquaculture. For instance, *Streptococcus iniae* is one of typical pathogen causes of streptococcosis, which results in hemorrhagic septicemia and causes significant mortality in tilapia [2,3]. Over the past few decades, antibiotics or chemicals have been widely used in fish farming for prophylactic or therapeutic purposes, to avoid disease outbreaks. Unfortunately, the dissemination of drug resistant bacteria, food safety hazards and the impacts of microbial ecology derived from the abuse of antibiotics, have become global issues. Moreover, regular antibiotic use easily induced drug resistant bacteria in the fish gut and leads to gut dysbiosis, which may cause decreased nutrient metabolism and immunity [4]. Regarding these issues, the development of alternative disease control methods in aquaculture is necessary.

Probiotics, defined as live microbes that confer health benefits to hosts, have been considered ecofriendly alternatives to antibiotics as a strategy for disease biocontrol in aquaculture. Since Kozasa reported the first empirical application of probiotics in aquaculture in 1986 [5], a variety of bacterial species have been developed as probiotics and applied in aquaculture, and these species benefit hosts in various ways, such as providing hydrolytic enzymes to improve nutrient utilization and growth performance, enhancing the innate immunity of the host against pathogen infection and promoting the growth of beneficial microflora in the gut [6]. Unlike the mechanism of antibiotics in disease control, which is well known, the mechanism of probiotics involving the multiple benefits that contribute to host health is multifaceted and has not been comprehensively clarified. In general, probiotics are thought to compete with pathogens for adhesion sites and then produce metabolites (bacteriocin, organic acid and polysaccharides, etc.) in the gastrointestinal (GI) tract to suppress the growth of pathogens and activate the innate immune system of the host [7]. The defensive action of probiotics in fish, by regulating the immune system by cytokines, is considered one of the generally accepted mechanisms of probiotics. Cytokines secreted from immune cells, such as lymphoid cells, macrophages and monocytes, play critical roles in activating the innate immune responses of hosts against pathogenic infections. Thus, typical cytokine genes, such as interleukin-1β (IL-1β), IL-8 and tumor necrosis factor (TNF)-α, have been commonly used as biomarkers for viewing the immune status of fish hosts [8]. Moreover, the efficacy of probiotics in boosting the defense mechanism of the host is thought to be species or strain specific [9]. Thus, individual functional validation in a variety of aquatic animals is necessary to develop a new probiotic isolate in aquaculture.

*Bacillus* species are the most attractive organisms for a new probiotic in aquaculture, due to their sporulation response against harsh conditions. As feed supplements to fish, *Bacillus* species are nonpathogenic and nontoxic, as they produce antimicrobial substances against pathogenic infection and secrete hydrolytic enzymes to enhance nutrient utilization [10]. *Bacillus safensis*, which was first isolated as a contaminant from a spacecraft-assembly facility (SAF) at the Jet Propulsion Laboratory, USA, is a chemoheterotrophic bacterium with a high tolerance to salt, heavy metals and ultraviolet light [11]. *Bacillus safensis* is one of the most widespread species and is found in a wide range of habitats, including saline deserts, insect guts, plant bodies, human and animal excreta and soil [12]. Recently, Ficarra et al. [13] reported that, based on genome mining and random amplified polymorphic DNA (RAPD) analysis, *B. safensis* putatively encodes diverse hydrolytic enzymes, such as lipase, amylase, protease, xylanase, and pectinase. Subsequently, the properties of secreting exoenzymes from *B. safensis* were determined, revealing a huge commercial potential for biotechnological applications in diverse industries, including detergent, biofuel, food and fiber industries [14,15]. In animal husbandry, adding hydrolytic enzymes to animal feed positively effects growth, feed efficiency, immunity and gut microbiota by reducing nonstarch polysaccharide (NSP), and antinutritive factors have been reviewed [16]. However, applying the approach to aquaculture is less practical due to the increasing cost of adding enzymes to fish feed. Adding hydrolytic enzyme-producing probiotics in feed offers an alternative strategy to overcome the problem of high cost in aquaculture. Moreover, the properties of probiotic secreted hydrolytic enzymes also support the benefits of probiotics, as feed additives contribute to improving nutritional feed utilization, feed efficiency, fish growth and gut microbiota in aquaculture. For example, Nile tilapia fed protease and/or xylanase-producing probiotics had significantly increased endogenous digestive protease activity and feed efficiency, growth performance and gut microbiota were improved [17,18]. An amylase and protease-producing probiotic *B. subtilis* HAINUP40 was used as a feed supplement and significantly enhanced growth, innate immunity and disease resistance in Nile tilapia [19]. Rohu (*Labeo rohita*) fed fermented *Leucaena leucocephala* leaf meal by cellulase-producing *B. circulan* significantly enhanced growth performance [20]. These studies suggested that dietary supplementation with hydrolytic enzyme-producing probiotics is an effective approach for improving feed efficiency and degrading the antinutritional effect of NSP. Although the *Bacillus* genus has been widely used as a probiotic in aquaculture, to date, only one study has examined the use of *B. safensis* as a probiotic in aquaculture. The study reported by Zhang elucidated that dietary supplementation of fermented feed with mixed probiotics, including *B. safensis*, could improve the growth, immunity and intestinal flora of *Penaeus vannamei* [21]. In the present study, *Bacillus safensis* NPUST1 was isolated from tilapia intestine as a potential probiotic with the ability to produce protease, amylase, cellulase and xylanase activities. The efficacy of dietary *B. safensis* on growth performance and nutrient utilization was evaluated based on growth parameters such weight gain (WE), feed efficiency (FE) and the expression of nutrient metabolism and growth-related genes. Innate immune parameters, including phagocytic activity (PA), respiratory burst (RB), superoxide dismutase (SOD) of head kidney leukocytes and serum lysozyme (*LYZ*) activity, and cytokine gene expression, were used to evaluate the efficacy of *B. safenesis* NPUST1 on the health status of tilapia. The efficacy of *B. safenesis* NPUST1 on disease resistance against pathogen infection was assessed by challenging tilapia with *S. iniae*. Moreover, the effect of *B. safenesis* NPUST1 on the intestinal microflora of Nile tilapia was analyzed by next-generation sequencing (NGS). These results could provide vital information in regard to the feasibility of *B. safensis* NPUST1 as a probiotic in aquaculture.

## 2. Materials and Methods

### 2.1. Fish Husbandry and Bacterial Strains

Nile tilapia (*Oreochromis niloticus*) fingerling was purchased from a local fish farm in Pingtung and reared with acclimation in 150-L glass aquaria containing 120 L of freshwater for 7 days in the Aquatic Laboratory Animals Facility of National Pingtung University of Science and Technology (NPUST), which was certified by the Association for Assessment and Accreditation of Laboratory Animal Care (AAALAC) International. Water quality was maintained by renewing about 30% of the rearing water 3 time a week. A temperature of 28 ± 1 °C, pH of 6.7 ± 0.2, and dissolved oxygen concentration of 6.1 ± 0.45 mg/L were maintained as the condition of water during acclimatization and feeding experimental period. The experimental protocols were carried out according to local animal welfare regulations and approved (approval No. NPUST-107-063) by the Institutional Animal Care and Use Committee (IACUC) of NPUST. The source and cultivation of the pathogen *S. iniae* were described in a previous report [22].

### 2.2. Isolation and Characterization of Potential Probiotics

Complete intestinal tissues were removed from six apparently healthy Nile tilapia (*O. niloticus*), which were collected from three domestic culture pools in Pingtung. The experiment for isolating potential probiotics was performed as described in a previous report [17]. Briefly, the individual intestine tissue was homogenized in sterile water and cultured in a 250-millileter flask containing 50 mL of TSB medium at 28 °C overnight to enrich the bacterial content. Five milliliters of the enriched culture were transferred to fresh TSB medium and incubated at 45 °C for 2 days to induce the formation of endospore-producing bacteria. Subsequently, the culture was shifted to an 80 °C water bath for 20 min to eliminate nonendospore-producing bacteria and was then spread and cultured on TSB agar medium at 28 °C for 24 h. Each colony was screened to assay the extracellular protease, amylase, cellulase and xylanase activity by an agar-well diffusion test. Briefly, wells 6 mm in diameter were created individually on differential agar plates. A 30 μL aliquot of each isolate was added to each well and incubated at 28 °C for 24 h. For the protease activity assay, the isolates were inoculated on 0.5% skimmed milk enriched nutrient agar plates, and the clear zones on the plates indicated protease activity. For the amylase activity assay, bacterial isolates were inoculated on 0.2% starch enriched nutrient agar plates, and amylase activity was observed by the clear zones around the wells after staining the culture plates with 5% iodine solution. For the cellulase and xylanase activity assay, bacterial isolates were inoculated separately on 0.5% carboxymethyl cellulose (CMC) and 1% xylan enriched nutrient agar plates, and cellulase activity was observed by the clear zones around the wells after the culture plates were stained with 0.4% Congo red solution. An isolate with multiple powerful hydrolytic enzyme activities was isolated as a potential probiotic. The identification of potential probiotics was performed by the Bioresource Collection and Research Centre (BCRC) using 16S rDNA sequencing and biochemical analysis. The sequences of the amplified 16S rDNA were blasted against known sequences in GenBank using Basic Local Alignment Search Tool (BLAST) (NCBI Nucleotide BLAST. https://blast.ncbi.nlm.nih.gov/Blast.cgi, accessed on 30 November 2021) and the phylogenetic tree was constructed by using the molecular evolutionary genetics analysis (MEGA) 4.1 software (available online: http://www.megasoftware.net/, accessed on 30 November 2021). The biochemical properties of potential probiotics were determined by typical microbiology tests, including Gram staining, catalase tests, oxidase tests, motility tests, enzyme activity tests and analyses on the fermentation ability of diverse carbohydrates [11].

### 2.3. Diet Preparation

The protocol for preparing diets was modified in accordance with a previous report [22]. Briefly, *B. safenesis* NPUST1 was cultured with 100 mL of TSB medium in an incubation shaker at 28 °C at 175 rpm for 16 h. After incubation, the culture broth was centrifuged at 6000× *g* for 15 min at 4 °C to harvest the bacterial cells. The bacterial pellet was washed three times with phosphate buffered saline (PBS) and then resuspended in sterile water at a concentration of 1 × 10^9^ CFU/mL. The ingredient and proximate compositions of the experimental diets are presented in Table 1. The proximate compositions of the experimental diet were determined by the AOAC method [23]. An appropriate amount of suspended bacterial cells was added to the ingredients of the basal diet to prepare a diet containing 10^5^ colony-forming units (CFU)/g, 10^6^ CFU/g and 10^7^ CFU/g *B. safensis* NPUST1. The diet was prepared by thoroughly mixing the ingredients, which were ground in a hammer mill and passed thoroughly through an 80-mesh screen (150 μm) before use. The mixed ingredients formed a stiff dough in a mixer, and then the dough was passed through a food grinder (die diameter of 3 mm), resulting in pellets with a particle size of approximately 1 cm. The pellets were dried in a drying cabinet using an air blower at 37 °C until the moisture content was approximately 10% and then stored in plastic bins at 4 °C until use. The viability of probiotic cells in the diet was monitored by performing plate counting on TSB agar plates every two weeks during the feeding trial.

### 2.4. Feeding Trail

A total of 360 juvenile Nile tilapia (*O. niloticus*) with an average body weight (BW) of 0.55 ± 0.042 g were randomly divided into control, G1, G2 and G3 groups and cultured. Each group was performed in triplicate in a 120-liter tank (*n* = 30/tank) with an independent recirculation system. The control group fish were fed a basal diet. The fish in the G1, G2 and G3 groups were fed a basal diet containing 10^5^ CFU/g, 10^6^ CFU/g and 10^7^ CFU/g *B. safensis* NPUST1, respectively. The fish were fed twice daily based on 5% body weight at 10:00 am and 4:00 pm. During the feeding period, the fish were weighed once every two weeks, and the amount of diet feed was adjusted accordingly. Each tank was cleaned by siphoning the water daily, and uneaten feed was gathered 1 h after each feeding to determine the feed intake. After 8 weeks of feeding, the growth parameters, digestive enzyme activities, innate immune parameters and gene expression, challenge test and gut microbiota were determined.

### 2.5. Growth Performance Parameters

The weights of all tilapia from each tank were measured every two weeks to determine the growth parameters, including weight gain (WG), specific growth rate (SGR), feed efficiency (FE) and survival rate (SR). The calculation was performed according to the following equations: WG = final BW − initial BW; SGR = (ln final BW−ln BW) × 100/duration of the experiment in days; FE = (final BW−initial BW)/feed intake; SR= 100 × (final number of test fish)/(initial number of test fish).

### 2.6. Assessment of the Digestive Enzyme Activities in Gut

Six fish from each group were sacrificed and dissected to sample their intestinal tissue at the end of the feeding trial. Two hundred milligrams of intestinal tissue from each fish was homogenized in 1 mL of chilled PBS buffer and then centrifuged at 6000× *g* for 10 min at 4 °C. The supernatant was then transferred to a 1.7 mL centrifuge tube and placed on ice for quantitative assays of digestive enzyme activities. The total protein of the suspension was measured by the Bradford method [24]. The digestive protease, amylase, cellulase and xylanase activities of the tilapia intestine were analyzed in accordance with a previous report [17].

### 2.7. Assessment of the Innate Immune Parameters

The efficacy of potential probiotic *B. safensis* NPUST1 on immune modulation was assessed by phagocytic activity (PA), respiratory burst (RB) activity and superoxide dismutase (SOD) activity of the head kidney, and lysozyme activity of serum. Six fish from each group were sampled at the end of the feeding trial. The procedures for harvesting blood samples and leukocytes of the head kidney and for conducting PA, RB activity, SOD activity and lysozyme activity followed a previously described protocol [22]. Total RNA from tilapia head kidney and spleen was extracted using TriPure isolation reagent (Roche, Mannheim, Germany) according to the manufacturer’s protocol. The cDNA from 1 μg of total RNA was generated using an iScript cDNA Synthesis Kit (Bio-Rad, Hercules, CA, USA). The expression levels of indicator genes involved in antioxidative stress and innate immunity were determined by quantitative PCR (qPCR). The expression of elongation factor (EF)-1α was used as an internal control. The specific primers used for determining each gene are listed in Table 2. The qPCR was conducted using the KAPA SYBR FAST qPCR Master Mix Kit (KAPA KR0389, Wilmington, MA, USA), and the amplification program was carried out as follows: 60 °C for 2 min, 95 °C for 10 min, and 40 cycles of denaturing at 95 °C for 15 s; annealing and primer extension at 60 °C for 1 min. The relative expression level of each gene normalized to EF-1α expression was expressed as the mean ± standard error (SE).

### 2.8. Challenge Experiment

The pathogen *S. iniae* was cultured in TSB medium and incubated at 28 °C for 24 h. The bacterial cells were collected by centrifuging at 6100× *g* for 15 min at ambient temperature and were then resuspended in an appropriate volume of distilled water. The bacterial challenge test was conducted in triplicate by performing an intraperitoneal (I.P.) injection of 50 µL of diluted *S. iniae* into the fish at a concentration of 10^5^ CFU per gram of body weight. Experimental fish (10 fish per tank) were kept in a 60 L tank containing 40 L of fresh water at 28 ± 1 °C. Fish fed the control diet that were injected with saline were used as the negative control, and each group was tested in triplicate. The challenged fish were observed daily, and cumulative survival was recorded for 7 days.

### 2.9. Intestinal Microbiota Analysis

At the end of the feeding trail, whole intestines of tilapia from each group (four samples from each group) were sampled, cut open, and rinsed with sterilized water to remove the intestinal contents. Microbial genomic DNA from each intestinal tract was extracted using an EasyPure Genomic DNA Spin Kit according to the manufacturer’s instructions. The concentration of extracted genomic DNA was determined by a Nano300 Micro-Spectrophotometer (Medclub Scientific Co., Ltd., Taoyuan City, Taiwan) and adjusted to 1 ng/µL to amplify the 16S gene. The full length 16S gene (V1–V9 regions) was amplified by PCR using 5′ phosphate modification and barcode degenerated 16S-specific forward primer 5′-Phos/GCATCAGRGTTYGATYMTGGCTCA-3′ and reverse primer 5′-Phos/GCATCRGYTACCTTGTTACGACTT-3′. The degenerate base identities included the following: R = A, G; Y = C, T; M = A, C. The PCR was carried out with KAPA HiFi HotStart ReadyMix (Roche) under the following PCR condition: 95 °C for 3 min; 20–27 cycles (sample dependence) of 95 °C for 30 s, 57 °C for 30 s, and 72 °C for 60 s; 72 °C for 5 min then maintain at 4 °C. The PCR products (1500 bp) were monitored on a 1% agarose gel and purified by using AMPure PB Beads for library preparation. Library construction was prepared using a Full-Length 16S Amplicication SMRTbell Library Preparation and Sequencing Kit (PacBio) following the manufacturer’s protocol. Sequencing was performed in circular consensus sequence (CCS) mode on a PacBio Sequel IIe system to generate HiFi reads with a minimum predicted accuracy of 0.9. After demultiplexing, the CCSs were further processed with DADA2 [25] to obtain amplicons with a single nucleotide resolution. The DADA2 algorithm resolves exact amplicon sequence variants (ASVs) with single nucleotide resolution from the full length 16S rRNA gene with a near zero error rate. Principal coordinate analysis (PCoA) was performed using the distance matrix to acquire principal coordinates for visualization of sophisticated and multidimensional data [26]. For each representative sequence, the feature-classifier and classify-consensus-blast algorithms in QIIME2 [27] were employed to annotate taxonomy classification based on the information retrieved from the NCBI database.

### 2.10. Statistical Analysis

A one-way ANOVA was used to analyze the data. When ANOVA identified differences among the groups, Tukey’s multiple comparison test was conducted to examine significant differences (*p* < 0.05) among the treatments. Cumulative survival in the challenge experiment was analyzed by the Kaplan–Meier method. Data were analyzed using SAS software (SAS Institute, Cary, NC, USA).

## 3. Results

### 3.1. Isolation of the Potential Probiotics with Hydrolytic Enzyme Activities

**Figure 1:**
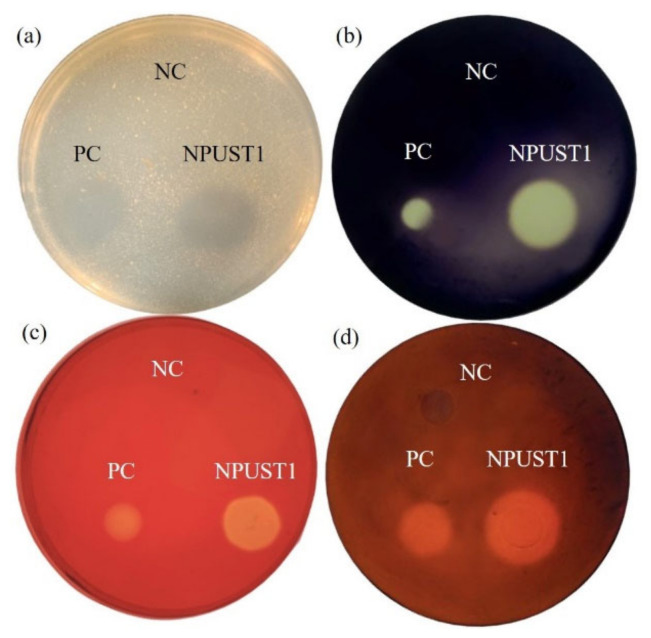
Analysis of digestive enzymes activities of NPUST1 by selective agar plate after cultivating for 48 h. (**a**) Protease activity; (**b**) amylase activity; (**c**) cellulase activity; (**d**) xylanase activity. *Escherichia coli* used as negative control (NC); *Bacillus amyloliquefaciens* R8 used as positive control (PC).

**Figure 2:**
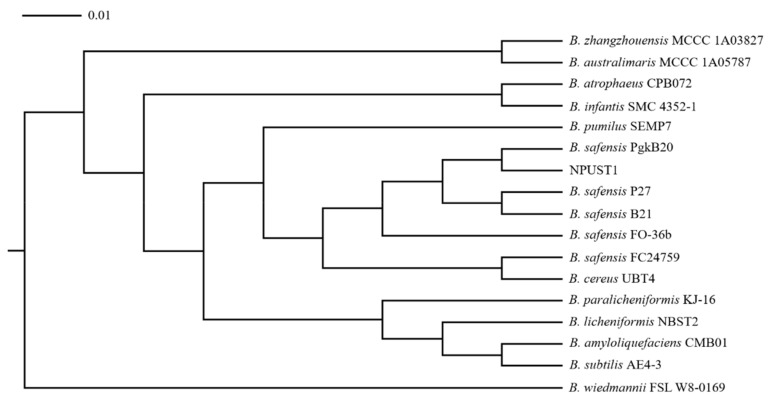
Phylogenetic tree of strain NPUST1 based on the 16S rDNA sequences. The 16S rDNA sequences of *Bacillus* strains were obtained from NCBI GenBank database (accession numbers are as follows: *B. zhangzhouensis* MCCC 1A03827, JX680133; *B. australimaris* MCCC 1A05787, JX680098; *B. atrophaeus* CPB072, KF751643; *B. infantis* SMC 4352-1, AY904032; *B. pumilus* SEMP7, JX915819; *B. safensis* PgkB20, MF979091; *B. safensis* P27, MN092366; *B. safensis* B21, MK499471; *B. safensis* FO-36b, NR_041794; *B. safensis* FC24759, MK577399; *B. cereus* UBT4, KR709243; *B. paralicheniformis* KJ-16, KY694465; *B. licheniformis* NBST2, GU011947; *B. amyloliquefaciens* CMB01, AF489591; *B. subtilis* AE4-3, AB269766; *B. wiedmannii* FSL W8-0169, KU198626).

Intestinal tissues from six Nile tilapia were sampled to isolate the potential probiotics according to the protocol described in Section 2.2 of the Material and Methods. A total of 86 isolates were selected based on morphological features and were further cultured on selective agar plates for screening potential probiotics. One strain, NPUST1, with intensive protease, amylase, cellulase, and xylanase activities was isolated (Figure 1). The identity of the NPUST1 was genotypically characterized by 16S rDNA sequencing and biochemical analysis. Biochemical properties revealed that NPUST1 was a gram-positive, endospore-forming, rod shaped, motile aerobic bacteria with catalase, β-galactosidase and gelatinase activities and possessed the ability to ferment diverse carbohydrates, including L-arabinose, D-ribose, D-xylose, D-galactose, D-glucose, D-fructose, D-mannose, D-mannitol, D-cellobiose, D-maltose, D-saccharose, D-trehalose, and D-tagatose. The 16S rDNA sequence of NPUST1 was deposited into GenBank (Accession number OL477428). Based on the results of 16S rDNA sequencing, NPUST1 was found to share 99% similarity to *Bacillus safensis* PgKB20 (Accession number CP043404.1) based on the BLAST results against known bacterial 16S rDNA sequences from the NCBI GenBank database. A phylogenetic was constructed with 16S rDNA sequences and demonstrated that the selected NPUST1 strain belonged to *Bacillus safensis* (Figure 2).

### 3.2. B. safensis NPUST1 Supplementation Enhances Nutrient Utilization and Growth Performance

**Figure 3:**
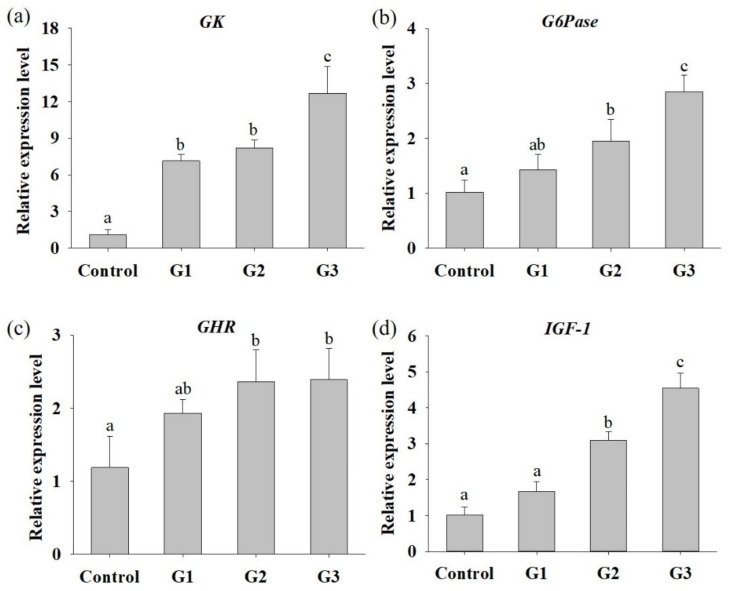
Relative expression levels of hepatic genes that participated in glucose metabolism and growth in Nile tilapia fed with basal diet (control) and basal diet containing 10^5^ (G1), 10^6^ (G2) or 10^7^ (G3) CFU/g of *B. safensis* NPUST1 for 8 weeks. (**a**) Glucokinase (*GK*), (**b**) glucose-6 phosphatase (*G6P*), (**c**) growth hormone receptor (*GHR*), and (**d**) insulin-like growth factor (*IGF-1*). The results represent means ± S.D. of six fishes per group. Bars with different superscripts are significantly different (*p* < 0.05, one-way ANOVA, Tukey’s multiple comparisons test).

To assess whether *B. safensis* NPUST1 can effectively enhance growth performance, parameters including WG, FE and SGR were evaluated after feeding *B. safensis* NPUST1 for 8 weeks. As shown in Table 3, the survival rate between each group was not significantly different, indicating that *B. safensis* NPUST1 is harmless to Nile tilapia. The WGs in the G2 and G3 group were 0.87 ± 0.114 g and 1.02 ± 0.111 g, respectively, which were significantly higher than the WGs in the G1 group (0.71 ± 0.025 g) and control group (0.75 ± 0.020 g). Similarly, the SGR and FE in Nile tilapia of the G2 and G3 groups were significantly higher than those of the G1 and control group fish. There were no significant differences in WG, FE or SGR in tilapia between the G1 and control groups or between the G2 and G3 groups. The improved FE of tilapia in the G2 and G3 groups prompted us to further study the hydrolytic enzyme activities in the fish gut and nutrient metabolism at the molecular level in the liver. As shown in Table 4, although there was no significant difference in xylanase activity between each group of tilapia, intestinal protease, amylase and lipase activities were significantly increased in the G2 and G3 group fish compared to those in the control group fish. Cellulase activity was significantly increased in the G3 group fish compared to that in control group. The mRNA expression levels of hepatic *GK*, *G6Pase*, *GHR* and *IGF-1* were significantly increased in the G2 and G3 group tilapia compared to the control group fish (Figure 3). Moreover, the expression of *GK* and *G6Pase* was also significantly increased in the G3 group compared to that of the G2 group. The increased mRNA expression of glucose metabolism and growth-related genes supports the results of the growth performance feeding trial. These results suggested that dietary supplementation of *B. safensis* NPUST1 with 10^6^ and 10^7^ CFU/g could improve nutrient utilization and growth performance.

### 3.3. B. safensis NPUST1 Supplementation Enhances Innate Immunity

**Figure 4:**
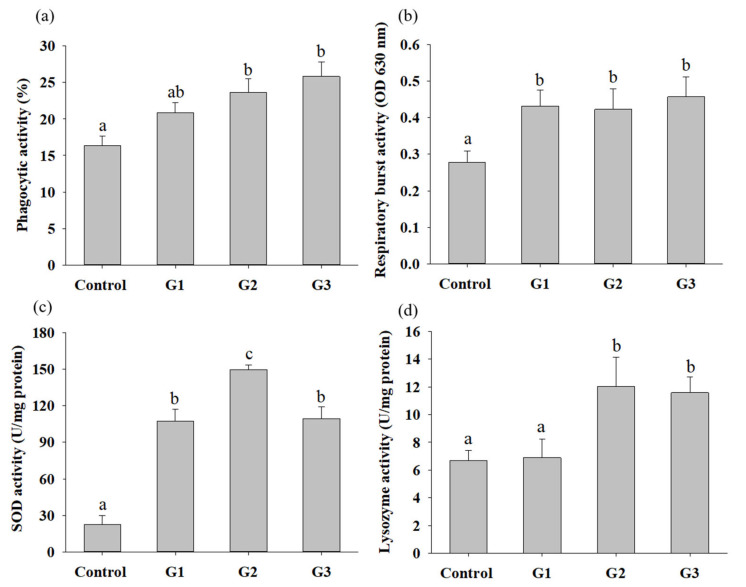
Innate immune responses of Nile tilapia after being fed a basal diet (control) and basal diet containing 10^5^ (G1), 10^6^ (G2) or 10^7^ (G3) CFU/g of *B. safensis* NPUST1 for 8 weeks. (**a**) Phagocytic activity, (**b**) respiratory burst activity, (**c**) SOD activity and (**d**) lysozyme activity. The results represent means ± S.D. of six fishes per group. Bars with different superscripts are significantly different (*p* < 0.05, one-way ANOVA, Tukey’s multiple comparisons test).

**Figure 5:**
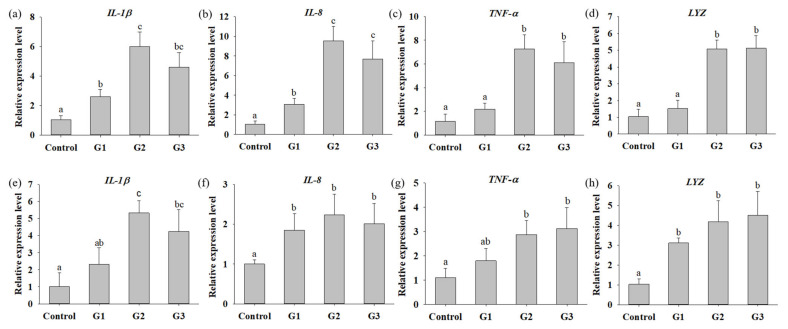
Quantitative PCR analysis of immune related expression in Nile tilapia fed with basal diet (control) and basal diet containing 10^5^ (G1), 10^6^ (G2) or 10^7^ (G3) CFU/g of *B. safensis* NPUST1 for 8 weeks. Expression of *IL-1β*, *IL-8*, *TNF-α*, and *LYZ* in head kidney (**a**–**d**) and in spleen (**e**–**h**). The results represent means ± S.D. of six fishes per group. Bars with different superscripts are significantly different (*p* < 0.05, one-way ANOVA, Tukey’s multiple comparisons test).

To understand whether supplementation with *B. safensis* NPUST1 can effectively enhance innate immunity, immune parameters, including the phagocytic activity (PA), respiratory burst (RB) activity and superoxidase dismutase (SOD) activity of head kidney leukocytes and serum lysozyme activity, were evaluated. The PA and lysozyme activities in the G2 and G3 group tilapia were significantly increased compared with those in the control group fish. There was no significant difference in the PA of fish between the G2 and G3 groups (Figure 4a,d). The respiratory burst activity and SOD activity in fish fed a diet containing *B. safensis* NPUST1 were also significantly higher than those in the control group fish (Figure 4b,c). Moreover, the SOD activity of fish in the G2 group was significantly increased compared with that of the G1 and G3 group fish (Figure 4c). The effect of *B. safensis* NPUST1 on innate immunity was investigated at the molecular level by determining the expression of the cytokine genes *IL-1β*, *IL-8*, *TNF-α* and *LYZ* in the head kidney and spleen. The expression of *IL-β* and *IL-8* in the head kidney significantly increased in fish fed the diet containing *B. safensis* NPUST1, compared to that of fish in the control group. Moreover, the expression of *IL-β* and *IL-8* in the G2 group fish was significantly higher than that in the G1 group fish (Figure 5a,b). The expression of *TNF*-*α* and *LYZ* in the head kidney was significantly increased in the G2 and G3 group fish compared to that of the control and G1 group fish (Figure 5c,d). Similarly, the expression of *IL-1β*, *IL-8*, *TNF-α* and *LYZ* in the spleen was significantly higher in the G2 and G3 group fish than in the control group fish (Figure 5e–h). In addition, the expression of *IL-8* and *LYZ* in the spleen of the G1 group fish was also significantly higher than that of the control group fish (Figure 5f,h). In summary, the results suggested that fish fed a *B. safensis* NPUST1 supplemented diet have immunomodulatory functions. Moreover, increased immune parameters and cytokine gene expression were commonly exhibited in the G2 and G3 group fish without significant differences, suggesting that a dose of 10^6^ CFU/g *B. safensis* NPUST1 was enough to enhance innate immunity.

### 3.4. Dietary B. safensis NPUST1 Enhances Defense against S. iniae Infection in Tilapia

**Figure 6:**
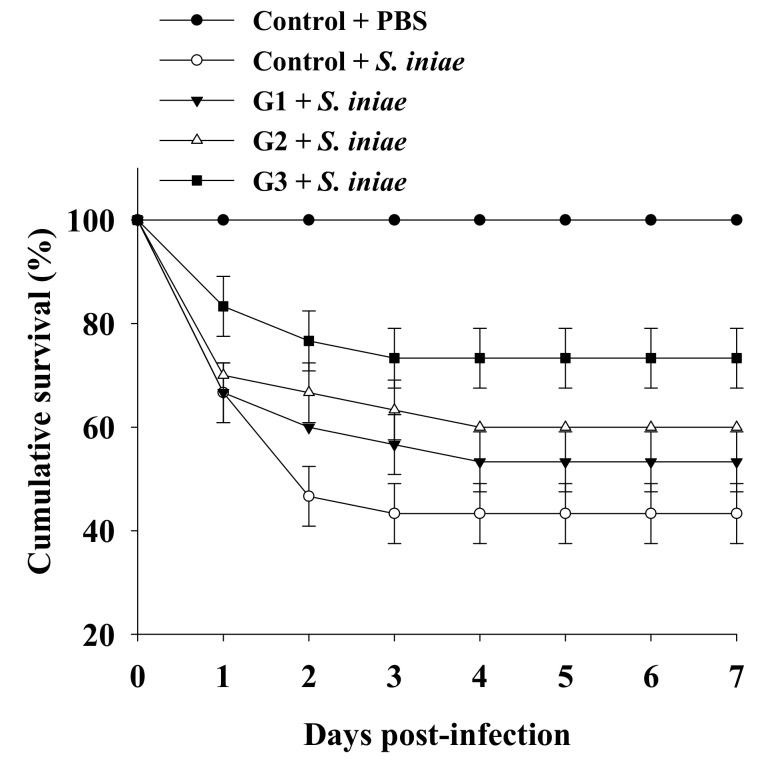
Cumulative survival of Nile tilapia challenged with *S. iniae* after being fed a basal diet (control) and basal diet containing 10^5^ (G1), 10^6^ (G2) or 10^7^ (G3) CFU/g of *B. safensis* NPUST1 for 8 weeks. Data are presented as the mean ± S.E. from triplicates of each group. The survival in the G2 and G3 groups was significantly higher than that in the control group based on the Kaplan–Meier method (*p* < 0.05).

The significantly increased innate immunity of tilapia fed a *B. safensis* NPUST1 supplemented diet prompted us to investigate whether the immunomodulatory function of *B. safensis* NPUST1 could be reflected in disease resistance, which was determined by measuring the survival rate of tilapia fed a diet containing *B. safensis* NPUST1 after challenge with *S. iniae*. The survival rate was maintained at 100% for the group injected with PBS buffer for 7 days postinfection. Conversely, the cumulative survival rate in the control group injected with *S. iniae* obviously declined during the first 2 days postinfection, and then remained at 43 ± 5.7% until 7 days postinfection. Although the cumulative survival in the G1 group injected with *S. iniae* was slightly higher than that in the control group injected with *S. iniae*, there was no significant difference in cumulative survival between the groups. Notably, the cumulative survival was 60% and 73 ± 5.7% in tilapia of the G2 and G3 groups, respectively, which was significantly increased compared to that in the control group fish. There was no significant difference in cumulative survival between the G2 and G3 groups (Figure 6). These results suggested that tilapia fed a *S. safensis* NPUST1 supplemented diet experienced disease resistance against *S. iniae* infection, and a dose of 10^6^ CFU/g of *B. safensis* NPUST1 was sufficient to acquire the function of disease resistance.

### 3.5. Dietary B. safensis NPUST1 Improve Intestinal Microbiota

**Figure 7:**
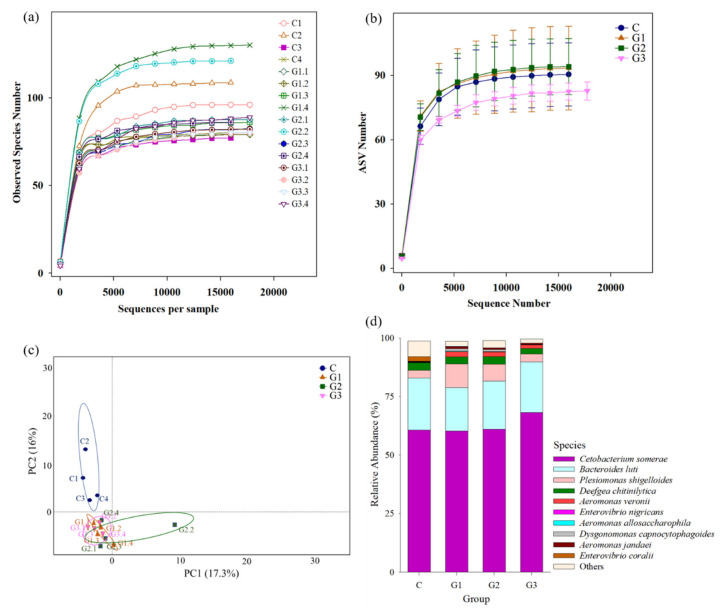
Comparison of gut microbiota composition between tilapia fed basal diet (control) and basal diet containing 10^5^ (G1), 10^6^ (G2) or 10^7^ (G3) CFU/g of *B. safensis* NPUST1. (**a**) Rarefaction analysis of each sample. (**b**) The rarefaction curve of the gut microbiota from fish in diverse groups. (**c**) Principal component analysis (PCA) of the compositions of microbes in the intestines of fish between different groups. (**d**) Relative abundances of different bacterial species in the intestines of fish between different groups.

The effects of *B. safensis* that altered the gut microbiota were analyzed by high throughput NGS. A total of 259,936 validated nucleotide sequences were retrieved from the gut content of 16 fish from the four groups (*n* = 4 for each group). The validated nucleotide sequences were, thereafter, assigned to 465 ASVs based on a 97% similarity level using the QIIME pipeline and plotted in a rarefaction curve. The sample rarefaction curve tended to approach a plateau, indicating that sufficient sampling depth was performed in each group (coverage reached 99.9%) (Figure 7a,b). The relationship of gut microbiota composition in each group was analyzed by PCA. The gut microbiota composition of *B. safensis* NPUST1 fed fish in G1, G2 and G3 were clustered closely and were separated from that in the control group, suggesting that supplementation with *B. safensis* NPUST1 impacted the microbiota composition of fish (Figure 7c). The most abundant species in Nile tilapia of each group was *Cetobacterium somerae*, which made up 60.72%, 60.28%, 61.05% and 68.19% of reads in the control, G1, G2 and G3 groups, respectively. The three predominant species among the four groups were the same: *Bacteroides luti*, *Plesiomonas shigelloides*, and *Deefgea chitinilytica*. The relative abundances of *Bacteroides luti* in the control, G1, G2, and G3 groups were 22.23%, 18.58%, 20.50%, and 21.69%, respectively; *Plesiomonas shigelloides* in the control, G1, G2, and G3 groups was 3.20%, 6.03%, 5.27% and 4.45%, respectively; and *Deefgea chitinilytica* in the control, G1, G2 and G3 groups was 3.31%, 3.10%, 3.19% and 2.34%, respectively. There was no significant difference in the relative abundances of the four predominant species among the four groups (Figure 7d). In depth examination of bacterial species revealed significant alterations in relative abundances, and the putative probiotic and pathogen species of Nile tilapia and target probiotic *B. safensis* in the present study are listed in Table 5. The results showed that the *B. safensis* investigated in this study had higher abundances in the *B. safensis* NPUST1 supplemented groups than in the control group. The abundances of the putative probiotic species *B. licheniformis* and *Lactococcus lactis* in the bacterial community were significantly higher in G1, G2 and G3 than in the control group. Moreover, the putative pathogen species *Aeromonas veronii*, *A. jandaei*, *Enterovibrio nigricans* and *E. coralii* in the bacterial community were significantly lower in the *B. safensis* NPUST1 supplemented groups than in the control.

## 4. Discussion

Several reviews of applications of *Bacillus* species in aquaculture have been conducted, showing that they confer beneficial effects such as enhanced growth performance, improved gut microbiota, and modulated immunity against pathogen infections [10,28]. However, although a variety of *Bacillus* species have been used as probiotics in diverse fish species, a high proportion of *Bacillus* are derived from the non-natural habitats of the host, and only a few studies have involved extracellular digestive enzyme-producing *Bacillus*. A review reported that host derived probiotics are potentially considered to have the ability to colonize hosts and act as a part of the natural defense system [29]. Thus, isolating a putative probiotic from a natural host and validating its beneficial effects on natural hosts are necessary. In the present study, a putative probiotic, *B. safensis* NPUST1, with properties of secreting protease, amylase, cellulase and xylanase activities, was isolated from the gut of Nile tilapia, suggesting that *B. safensis* NPUST1 has the potential ability to colonize the tilapia intestinal epithelium and is safe for use in aquaculture.

Plant derived proteins, such as soybean meal, rice gluten and wheat middling, are commonly used to reduce the use of fish meal in feed. Proteases are vital proteolytic enzymes that hydrolyze protein in food to liberate amino acids and can improve protein digestibility and absorptivity, resulting in enhanced feed efficiency and growth performance in fish. However, nonstarch polysaccharides (NSPs), which are indigestible carbohydrates in plant ingredients, are considered to have antinutritional value for fish by increasing the feed viscosity and decreasing the action of digestive enzymes [30]. Cellulose and xylan polysaccharides are the major components and are noncellulosic constituents in the cell wall of plants. Unfortunately, plant derived polysaccharides are difficult to effectively utilize because endogenous digestive cellulase and xylanase are less abundant in the GI tract of fish. Studies have reported that supplementation of hydrolytic enzymes such as cellulase and xylanase in feed can efficiently solve NSP problems in aquaculture [31,32]; however, the high cost of enzymes supplemented in feed is difficult to manage in aquaculture. Hence, supplementation with hydrolytic enzyme-producing probiotics is a practical strategy to reduce the antinutritional effects on nutrition utilization and growth performance. Reports have shown that dietary supplementation with hydrolytic enzymes (protease, amylase, cellulase and xylanase)-producing probiotics could obviously enhance FE and growth performance in white shrimp (*Litopenaeus vannamei*), zebrafish (*Danio rerio*), pla-mong (*Pangasius bocourti*), golden pompano (*Trachinotus ovatus*), and Nile tilapia [17,18,33,34,35,36]. These investigations showed that dietary hydrolytic enzyme-producing probiotics can promote fish growth by enhancing nutrition utilization. The present study showed that dietary supplementation with protease-, amylase-, cellulase- and xylanase-producing *B. safensis* NPUST1 significantly enhanced WG, FE and SGR in the G2 and G3 group fish, compared to those of the control group fish. Moreover, endogenous protease, amylase, cellulase and lipase were significantly increased in the GI tract of *B. safensis* NPUST1 supplemented fish. These results suggested that the mechanism of *B. safensis* NPUST1 contributes to growth enhancement in fish, probably due to the improvement of nutrition utilization, which was achieved by secreting hydrolytic enzymes and stimulating the production of digestive enzymes in the GI tract after *B. safensis* NPUST1 administration. The improved nutrient utilization and growth performance of fish fed a *B. safensis* NPUST1 supplemented diet were also reflected at the molecular level. The liver plays a critical role in glucose homeostasis and metabolism. Hepatic GK is a major hexokinase in vertebrates that is responsible for the phosphorylation of glucose to glucose-6-phosphate, which is the first step of glycolysis. The typical role of G6Pase in the liver is to release glucose into blood by catalyzing the final step of glycogenolysis. The growth hormone (GH)-IGF-I axis is a vital endocrine mechanism regulating growth in vertebrates. Hepatic GHR receives GH signals from the pituitary gland and thereby triggers the release of IGF-1 from the liver into the circulatory system to stimulate somatic growth. Dietary supplementation with protease- or xylanase-producing probiotics induced the expression of GK, G6Pase, GHR and IGF-1 in zebrafish and tilapia [18,35,36]. The present study showed that fish fed *B. safensis* NPUST1 had significantly increased expressions of GK, G6Pase, GHR and IGF-1, suggesting that hydrolytic enzymes (protease, amylase, cellulase and xylanase) producing *B. safensis* NPUST1 have a high capacity to improve growth performance by increasing nutrition utilization and metabolism.

Probiotics that contribute immunomodulatory functions to the host are considered to provide the most beneficial effects in aquaculture. The head kidney and spleen are major lymphoid organs of teleosts and play a vital role in regulating innate immunity against pathogenic invasion. Leukocytes such as neutrophils and macrophages in the head kidney of fish act bactericidally by recognizing invading pathogens directly or indirectly and then engulfing invading pathogens by phagocytosis. Respiratory bursts, which indicate highly bactericidal reactive oxygen species (ROS), such as superoxide anions (O_2_^−^), hydroxyl free radicals (OH^−^) and hydrogen peroxide (H_2_O_2_), are released from leukocytes to destroy engulfed pathogens during phagocytosis. Thus, PA and RB activity in leukocytes of the head kidney are critical indicators to judge the innate immunity of teleosts [37]. Fish lysozyme, which exhibits bactericidal activity by hydrolyzing peptidoglycan of the bacterial cell wall and restrains the growth of invaded pathogens, is an indispensable defense component of the innate immune system. In addition to its direct bactericidal activity, recent evidence has shown that lysozyme mediated bacterial lysates can modulate the innate immune response against pathogen infection by activating pattern-recognition receptors or the complement system [38]. In the present study, there was significantly increased PA and RB activity in lymphoid cells and serum lysozyme activity in the G2 and G3 group tilapia compared to those in the control group fish, suggesting that 10^6^ CFU/g and 10^7^ CFU/g of *B. safensis* NPUST1 supplementation in feed provides an immunomodulatory function in fish. SOD responds to oxidative stress by catalyzing the dismutation of superoxide anions (O_2_^−^) into hydrogen peroxide, which is commonly used as an indicator of protective ability against ROS damage. Reports have shown that dietary administration of *B. subtilis*, *Paenibacillus ehimensis* and *Rummeliibacillus staekisii* for 8 weeks increased the SOD activity in tilapia [17,19,22]. The present study showed that SOD activity significantly increased in the head kidney of tilapia fed *B. safensis* NPUST1 supplemented tilapia compared to that in the control group fish, suggesting that *B. safensis* NPUST1 modulates protective defense against oxidative stress.

IL-1β and TNF-α are typical proinflammatory cytokines that are induced at the early stage of pathogen infection to enhance macrophage survival and the bactericidal activity of leukocytes by increasing ROS production during phagocytosis. IL-8 is a well known chemotactic factor in fish, which attracts and activates neutrophils during the inflammatory process. Reports have shown that administration of the probiotics *Carnobacterium maltaromaticum*, *C. divergens*, *Lactobacillus acidophilus*, *L. lactis*, *L. plantarum*, *P. ehimensis*, *R. staekisii* significantly increased the expression of IL-1β, IL-8 and TNF-α in the head kidney leucocytes of rainbow trout (*Oncorhynchus mykiss* Walbaum) [39], common carp (*Cyprinus carpio*) [40], olive flounder (*Paralichthys olivaceus*) [41], and Nile tilapia (*O. niloticus*) [17,22]. The present study showed a significant increase in the expression of cytokine genes, including *IL-1β*, IL-8 and TNF-α, in the head kidney and spleen of tilapia of the G2 and G3 groups, compared to those in the control group tilapia. Moreover, the cumulative survival of tilapia challenged with *S. iniae* increased in the G2 and G3 tilapia, compared to the control tilapia, suggesting that supplementation with 10^6^ CFU/g and 10^7^ CFU/g *B. safensis* NPUST1 in feed can modulate innate immunity, subsequently contributing to improving protective defense against pathogen infection. In recent decades, diverse *Bacillus* species have been shown to exhibit immunomodulatory functions to enhance immunity against pathogen infections [10]. Nevertheless, no study has addressed *B. safensis* as a probiotic in aquaculture, thus far. The present study is the first report demonstrating that the use of *B. safensis* NPUST1 as a probiotic in aquaculture provides beneficial effects to fish.

The gut microbiota of fish has been shown to participate in the communication system of the gut–brain axis to mediate metabolism, immune responses and energy homeostasis [42]. Feed and feed additives have been considered one of the major factors affecting gut microbiota in fish. Reports have shown that probiotics as feed additives can effectively improve the bacterial community in the fish intestine, thereby further enhancing growth, innate immunity and resistance against pathogen infections in tilapia [17,43,44,45]. In the present study, the full length 16S rDNA sequences analyzed by NGS were used to examine the effects of *B. safensis* NPUST1 on the alteration of bacterial species in depth. The four predominant species in the tilapia intestine were *C. somerae*, *Bacteroides luti*, *Plesiomonas shigelloides* and *Deefgea chitinilytica*. The most predominant species, *C. somerae,* presented in this study is consistent with data revealed in reports that also showed that the *Cetobacterium* genus comprises the majority of the microbiome in Nile tilapia [17,46,47]. *Bacteroides* is the main genus in the lower intestinal tract, and molecular interactions among these species can influence the host’s immune system. Xia et al. [45] evidenced that *Bacterioides* sp. is one of the main components in the intestine of Nile tilapia supplemented with *B. creus* and *B. subtilis* probiotics. *Deefgea chitinilytica*, which belongs to the family Neisseriaceae, was recently isolated from goldfish (*Carassius auratus auratus*) [46]. However, studies on the physiological roles of *C. somerae*, *B. luti* and *D. chitinilytica* in Nile tilapia are still lacking and this needs to be further investigated in the future. Reports have shown that the *Plesiomonas* genus is also the predominant microbiome in the intestine of Nile tilapia [47]. *Plesiomonas shigelloides* is considered a potential pathogen in freshwater fish. Xia et al. [48] reported that supplementation with probiotics *Lactobacillus rhamnosus* and *L. lactis* significantly reduced *Plesiomonas* genus levels in tilapia. However, in the present study, there were no significant differences in the abundance of *Plesiomonas shigelloides* between each group, which may be due to the different probiotic species used in the study. Notably, the abundance of *B. safensis* significantly increased in the G1, G2 and G3 groups compared with the control group, suggesting that the fed *B. safensis* NPUST1 was actually already colonized in the fish intestines. *Aeromonas* species are considered pathogens in farmed freshwater fish, including tilapia. *A. veronii* and *A. jandaei* isolated from Nile tilapia have been confirmed to be virulent, and they cause severe disease and mortality [49]. Although few studies have reported the pathogenic relationship of the *Enterovibrio* genus and tilapia thus far, *E. corali* isolated from rainbow trout (*Oncorhynchus mykiss*) and *E. nigricans* isolated from torafugu (*Takifugu rubripus*) with high virulence have been identified as potential fish pathogens [50,51]. The present study showed that the potential probiotics *B. licheniformis* and *L. lactis* were more highly abundant while the potential pathogens *A. veronii*, *A. jandaei*, *E. nigricans* and *E. coralii* were less abundant in *B. safensis* NPUST1 supplemented fish, suggesting that *B. safensis* NPUST1 can improve the gut microbiota of Nile tilapia and provide beneficial effects on growth, immunity and disease resistance.

In conclusion, the present study isolated a hydrolytic enzyme (protease, amylase, cellulase, xylanase)-producing potential probiotic, *B. safensis* NPUST1, from the tilapia gut. Dietary administration of *B. safensis* NPUST1 in Nile tilapia for 8 weeks revealed enhanced growth, feed efficiency, innate immunity and disease resistance against *S. iniae*. Dietary supplementation with *B. safensis* NPUST1 provides beneficial effects that improve the gut microbiota. The improved gut microbiota contributes to immunity by increasing beneficial bacteria and decreasing harmful bacteria; however, the mechanism needs to be further explored in the future. The present investigation is the first report to study the effects of *B. safensis* as a probiotic, and the conclusion suggests that *B. safensis* NPUST1 can be used as a probiotic to enhance growth and health status in tilapia aquaculture.

## Figures and Tables

**Table 1:** Ingredients and proximate composition of the experimental diets (g k^−1^).

Ingredients	Treatment (g kg^−^^1^)			
	Control Group	G1 Group	G2 Group	G3 Group
*B. safensis* NPUST1	0	10^8^ CFU	10^9^ CFU	10^10^ CFU
Fish meal	50	50	50	50
Soybean meal	300	300	300	300
Wheat middling	161	161	161	161
Rice bran	300	300	300	300
α-starch	60	60	60	60
Cellulose	70	70	70	70
Skim milk	10	10	10	10
Soybean oil	30	30	30	30
Mineral mixture ^a^	16	16	16	16
Vitamin mixture ^a^	3	3	3	3
Proximate composition				
Crude protein	236.1	234.0	236.1	230.1
Crude lipid	103.5	104.8	102.1	101.6
Moisture	96.8	96.2	93.8	96.5
Ash	65.9	64.9	66.1	64.2

^a^ Mineral mixture (g kg^−1^ of mixture) and vitamin mixture (g kg^−1^ of mixture) purchased from the Everest Food Ingredients Laboratories Corp., Taichung, Taiwan.

**Table 2:** Primers used for detecting the expression level of specific genes in the present study.

Gene Name	Primer Sequences (5′→3′)	Amplicon Size	Accession No.
Glucose kinase (*GK*)	F: GCAGCGAGGAAGCCATGAAGA R: GAGGTCCCTGACGACTTTGTGG	101 bp	XM_003451020
Glucose-6-phosphatease (*G6 Pase*)	F: AGCGCGAGCCTGAAGAAGTACT R: ATGGTCCACAGCAGGTCCACAT	107 bp	XM_003448671
Growth hormone receptor-1 (*GHR-1*)	F: GAATACAAGTCCTTCCGGGCTAA R: CTCATACTCCACACGCATCCA	100 bp	AY973232
Insulin-like growth factor-1 (*IGF-1*)	F: TGTCTGCCAGTAAGGATGTTCTTG R: GGCTTTCCACGCCACTTAAC	100 bp	EU272149
Tumor necrosis factor (*TNF-α*)	F: CCAGAAGCACTAAAGGCGAAGA R: CCTTGGCTTTGCTGCTGATC	82 bp	AY428948
Interleukin-1β (*ΙL-1β*)	F: TGTCGCTCTGGGCATCAA R: GGCTTGTCGTCATCCTTGTGA	63 bp	KJ574402
Interleukin-8 (*IL-8*)	F: CCTCGAGAAGGTGGATGTGAA R: CATGAGACCCAGGGCATCA	100 bp	GQ355864
Lysozyme (*Lyz*)	F: GCCTGACCGAACATGAGTCA R: CACCAGCGGCTATTTATCTGAA	100 bp	LC012581
β-actin	F: CCACACAGTGCCCATCTACGA R: CCACGCTCTGTCAGGATCTTCA	111 bp	EU887951

**Table 3:** Growth performance of Nile tilapia (*O. niloticus*) after being fed a basal diet containing 10^5^, 10^6^ or 10^7^ CFU/g of *B. safens* is NPUST1 for 8 weeks.

Parameters	Administrations			
	Control	10^5^ CFU/g (G1)	10^6^ CFU/g (G2)	10^7^ CFU/g (G3)
Initial weight (g)	0.56 ± 0.021 ^a^	0.54 ± 0.016 ^a^	0.54 ± 0.020 ^a^	0.56 ± 0.017 ^a^
Finial weight (g)	1.31 ± 0.004 ^a^	1.25 ± 0.025 ^a^	1.41 ± 0.095 ^b^	1.58 ± 0.125 ^b^
Weight gain (g)	0.75 ± 0.020 ^a^	0.71 ± 0.013 ^a^	0.87 ± 0.114 ^b^	1.02 ± 0.111 ^b^
Feed efficiency	0.33 ± 0.010 ^a^	0.33 ± 0.015 ^a^	0.39 ± 0.044 ^b^	0.41 ± 0.034 ^b^
Specific growth rate	2.41 ± 0.153 ^a^	2.34 ± 0.061 ^a^	2.81 ± 0.471 ^b^	3.26 ± 0.290 ^b^
Survival rate (%)	97.8 ± 1.92 ^a^	95.6 ± 3.85^a^	96.7 ± 0.00 ^a^	97.8 ± 1.92 ^a^

Data are presented as the mean ± S.E. from triplicates of each group. Different superscripts in the same rows represent significant differences (*p* < 0.05, one-way ANOVA, Tukey’s multiple comparisons test).

**Table 4:** Intestinal digestive enzymes of Nile tilapia (*O. niloticus*) after being fed a basal diet containing 10^5^, 10^6^ or 10^7^ CFU/g of *B. safens* is NPUST1 for 8 weeks.

Activity (U/mg)	Control	G1	G2	G3
Protease	0.50 ± 0.007 ^a^	0.51 ± 0.009 ^a^	0.64 ± 0.013 ^b^	0.68 ± 0.005 ^b^
Amylase	0.15 ± 0.003 ^a^	0.15 ± 0.08 ^a b^	0.18 ± 0.004 ^b^	0.18 ± 0.009 ^b^
Cellulase	0.14 ± 0.010 ^a^	0.15 ± 0.005 ^a b^	0.16 ± 0.004 ^a b^	0.18 ± 0.025 ^b^
Xylanase	0.10 ± 0.001 ^a^	0.11 ± 0.013 ^a^	0.11 ± 0.007 ^a^	0.10 ± 0.001 ^a^
Lipase	0.03 ± 0.003 ^a^	0.05 ± 0.004 ^b^	0.05 ± 0.003 ^b^	0.06 ± 0.003 ^c^

Data are presented as the mean ± S.D. from 6 fishes per group. Different superscripts in the same rows represent significant differences (*p* < 0.05, one-way ANOVA, Tukey’s multiple comparisons test).

**Table 5:** Effects of *B. safensis* NPUST1 on significant changes in the bacterial species of interest.

Species	Control	G1	G2	G3
Potential probiotics				
*Bacillus safensis*	0.001%	0.082%	0.074%	0.091%
*Bacillus licheniformis*	0.002%	0.23%	0.19%	0.28%
*Lactococcus lactis*	0.004%	0.11%	0.09%	0.14%
Potential pathogens				
*Aeromonas veronii*	1.23%	0.12%	0.06%	0.07%
*Aeromonas jandaei*	0.95%	0.081%	0.017%	0.048%
*Enterovibrio nigricans*	0.31%	0.002%	0.005%	0.009%
*Enterovibrio coralii*	2.04%	0.01%	0.09%	0.007%

## Data Availability

The data presented in this study are available in the article.
